# Evaluation of gene expression in muscle in mouse model lacking of vitamin C synthesis

**DOI:** 10.1186/1471-2105-13-S12-A11

**Published:** 2012-07-31

**Authors:** Yonghui Ma, Yan Jiao, Fusheng Zhao, Weikuan Gu

**Affiliations:** 1 Department of Orthopedic Surgery – Campbell Clinic, University of Tennessee Health Science Center, Memphis, TN 38163, USA

## Background

Human species and guinea pigs have to obtain vitamin C (VC) from food because they are unable to synthesize ascorbic acid due to the absence of the gene that encodes L-gulonolactone oxidase (Gulo). The spontaneous bone fracture (sfx) mouse is a mouse model which is deficient in the synthesis of VC because of the deletion in Gulo gene. Because muscle forces are a strong determinant of bone structure, particularly during the process of growth and development, we examined the gene expression of muscle in sfx mice.

## Materials and methods

In order to identify the genes that regulate muscle development through Vitamin C (VC) pathway, we analyzed the gene expression profile in mouse muscle from femur. For microarray analysis, muscle from three age-matched, wild-type +/+ Balb/By, inbred strain mice (WT) and 3 female and 3 male homozygous sfx/sfx mice were used.

## Results

Our results indicated that 1) The expression of Myogenic factor 6 (Myf6) gene in sfx mice is increased in both female and male mice, while the increase in male mice is much higher than that in female mice. 2) Some of dystrophin relevant genes are also affected in the sfx mice. The expressions of adenylate kinase (Ak1), creatine kinase sarcomeric mitochondrial (Ckmt2), and calcium-and integrin-binding protein 2 (Cib2) are decreased. The expression of creatine kinase muscle type (Ckb) and Cdc42 binding protein kinase beta (Cdc42bpb), on the other hand, although at a low level are increased in both sfx and wild type mice. Pathological analysis suggests that the diameters of male and female of sfx mice are smaller than that of Wt mice.

Suggesting a deficiency in muscle development and potentially function.

## Conclusions

There is a significant difference in myopathy and gene regulations in muscle of sfx mice between female and male mice.

